# Laparoscopic adrenalectomy in resident training: ıs it safe and feasible?

**DOI:** 10.1590/1806-9282.20251029

**Published:** 2026-04-20

**Authors:** Mehmet Üstün, Olcay Atbaş, Göksever Akpınar, Korhan Tuncer, Ozan Barış Namdaroğlu, Sümeyye Ekmekci, Emel Ebru Pala

**Affiliations:** 1 Izmir University of Health Sciences, Tepecik Training and Research Hospital, Department of General Surgery – İzmir, Turkey.; 2 Bakırçay University, Çiğli Training and Research Hospital, Department of General Surgery – İzmir, Turkey.; 3 Izmir University of Health Sciences, Tepecik Training and Research Hospital, Department of Pathology – İzmir, Turkey.

**Keywords:** Adrenalectomy, Resident, Laparoscopy, Training

## Abstract

**OBJECTIVE:**

The aim of this study was to evaluate whether laparoscopic adrenalectomy can be safely performed by senior residents (4 years and above) with the guidance of senior surgeons.

**METHODS:**

Patients who underwent unilateral laparoscopic transabdominal adrenalectomy between January 2012 and December 2023 were enrolled. Patients were divided into two groups. Patients’ demographic, clinical, and surgical data were recorded and analyzed.

**RESULTS:**

A total of 106 patients were enrolled in the study. Twenty of the patients were male, and 86 were female. The mean age was 51.8 (±standard deviation±12.1). In group 1, there were 83 patients (78.3%) who were operated on by a senior surgeon, and in group 2, there were 23 patients (21.7%) who were operated on by a senior resident. There was no significant difference between the two groups in terms of age, gender, and American Anesthesia Society score. A statistically significant difference was detected between the two groups in terms of mass diameter (p=0.042). In multivariate analysis, being operated on by a senior surgeon was not a risk factor for either postoperative complications or conversion to open surgery.

**CONCLUSION:**

In conclusion, laparoscopic adrenalectomy can be safely performed by senior residents under the supervision of senior surgeons.

## INTRODUCTION

Traditional views have changed rapidly in many areas around the world in the last decade. Health services are also prone to this change. There are also changes in operating room safety, efficiency, working conditions of residents, and residents’ approach to surgical training, including the importance given to patient safety^1^.

Minimally invasive techniques became more popular and available and have therefore been incorporated into the training programs of surgical residents. Procedures such as cholecystectomy and appendectomy, where the laparoscopic method became the gold standard, have taken their place in general surgery resident training. It's now discussed whether advanced laparoscopic procedures can also be performed by residents as safely as by senior surgeons.

Laparoscopic adrenalectomy (LA) is one of these advanced surgical procedures and has become the gold standard for the majority of adrenal resections today^2^.

This study aims to evaluate whether laparoscopic adrenalectomy can be safely performed by senior residents (4 years and above) with the guidance of an experienced surgeon.

## METHODS

Patients who underwent unilateral laparoscopic transabdominal adrenalectomy between January 2012 and December 2023 were enrolled. All patients were operated on with the transabdominal approach. Exclusion criteria were patients undergoing open adrenalectomy, adrenalectomy as a part of multiorgan resection, bilateral adrenalectomy, or adrenalectomy from a posterior approach. Patients were divided into two groups, operated on by the senior surgeon and senior resident (4 years and above). All surgical procedures were attended by a senior surgeon, whether acting as the first surgeon or as the tutor of the operating resident.

Demographics, history of previous surgery, ASA (American Anesthesia Society) score, mass diameter (mm), side, operation length (minutes), hospital stay (days), histopathology result, perioperative complications, postoperative complications, and mortality were compared between the two groups.

Approval from the institutional research ethics board was obtained (decision number 2024/08-09).

### Statistical analysis

Statistical analyses were performed using SPSS (Statistical Package for the Social Sciences [IBM Corp., Armonk, NY, USA]) version 25.0 software. The conformity of variables to normal distribution was examined using analytical methods (Kolmogorov-Smirnov/Shapiro-Wilk tests). Descriptive analyses were given using mean±standard deviation for normally distributed variables and median (Q1–Q3) for those not normally distributed. Descriptive statistics were performed by giving demographic characteristics, frequency, and percentage values. In continuous data, the t-test was used for independent groups showing normal distribution, and the Mann-Whitney U test was used for those not normally distributed. Pearson chi-square or Fisher's exact chi-square test was used in the analysis of categorical data. Univariate analysis was performed to find potential risk factors, and then multivariate analysis was performed to identify independent risk factors. In multivariate logistic regression analysis, variables were selected based on both statistical significance in univariate analysis and clinical significance supported by the literature. All predefined variables considered clinically significant (age, gender, previous abdominal surgery, procedure performed by resident, operation length, mass diameter, mass location, and laparoscopic/open approach) were included in the multivariate model. This approach was chosen to adjust for potential confounders and to investigate the independent impact of each factor on postoperative complications despite the limited number of outcome events. A p<0.05 value was considered statistically significant.

## RESULTS

A total of 106 patients were enrolled in the study. Twenty of the patients were male, and 86 were female. The mean age was 51.8 (±standard deviation [SD]±12.1). The patients were comparatively evaluated in two groups. In group 1, there were 83 patients (78.3%) who were operated on by a senior surgeon, and in group 2, there were 23 patients (21.7%) who were operated on by a senior resident (4 years and above). There was no significant difference between the two groups in terms of age, gender, and ASA score. There was previous abdominal surgery in nine patients (10.8%) in group 1 and three patients (13%) in group 2 (p=0.720). The median mass diameter of patients in group 1 was 65 mm (55–80), and the median mass diameter of patients in group 2 was 60 mm (33–70). A statistically significant difference was detected between the two groups in terms of mass diameter (p=0.042). When the mass localization was examined, 35 (42.2%) of the patients in group 1 were on the right side, and 48 (57.8%) were on the left side; 9 (39.1%) of the patients in group 2 were on the right side, and 14 (60.9%) were on the left side. Conversion to open surgery was performed in 10 patients (12%) in group 1 and in one patient (4.3%) in group 2 (p=0.450). Other organ injury was detected in one patient, and massive bleeding was detected in one patient in group 1. No perioperative complications developed in any patient in Group 2. There were postoperative complications in eight patients (9.6%) in group 1 and four patients (17.4%) in group 2. No statistically significant difference was found in both groups (p=0.287). The median length of stay in group 1 was 2 days, while it was 4 days for patients in group 2. This was statistically significant between the two groups (p<0.001). Clinicopathological data between the two groups are given comparatively in Table 1, showing no significant difference between groups.

**Table 1:** Comparison of demographic, clinical, and operative characteristics between senior surgeon and resident groups.

			Total patients	Senior	Resident	p-value
			n=106	n=83	n=23	
Age, mean±SD			51.8±12.1	52.3±12.3	49.9±11.8	0.414
Sex, n (%)						0.765*
	Male		20 (18.9)	15 (18.1)	5 (21.7)	
	Female		86 (81.1)	68 (81.9)	18 (78.3)	
ASA score, n (%)						0.159
	ASA 2		64 (60.4)	51 (61.4)	13 (56.5)	
	ASA 3		41 (38.7)	32 (38.6)	9 (39.1)	
	ASA 4		1 (0.9)	0	1 (4.3)	
Previous surgery, n (%)			12 (11.3)	9 (10.8)	3 (13)	0.720*
Open surgery, n (%)			11 (10.4)	10 (12)	1 (4.3)	0.450*
Mass diameter (mm), median (Q1–Q3)			65 (54.8–75)	65 (55–80)	60 (33–70)	0.042
Localization, n (%)						0.794
	Right		44 (41.5)	35 (42.2)	9 (39.1)	
	Left		62 (58.5)	48 (57.8)	14 (60.9)	
Operation length, median (Q1-Q3)			150 (125–181)	145 (125–180)	165 (140–185)	0.081
Peroperative complications, n (%)						1.000*
	Non-peroperative complications		104 (98.1)	81 (97.6)	23 (100)	
	Having a perioperative complication		2 (1.9)	2 (2.4)	0	
		Tumor capsule rupture	–	–	–	
		Injury to other organs	1 (0.9)	1 (1.2)	–	
		Massive bleeding	1 (0.9)	1 (1.2)	–	
Histopathology, n (%)						0.294
	Adrenocortical adenoma		61 (57.5)	45 (54.2)	16 (69.6)	
	Pheochromocytoma		20 (18.9)	18 (21.7)	2 (8.7)	
	Lipoma		11 (10.4)	9 (10.8)	2 (8.7)	
	Adrenocortical cyst		6 (5.7)	5 (6)	1 (43)	
	Hamartom		1 (0.9)	1 (1.2)	0	
	Adrenocortical carcinoma		2 (1.9)	1 (1.2)	1 (4.3)	
	Metastasis		4 (3.8)	4 (4.8)	0	
	Adrenocortical oncocytoma		1 (0.9)	0	1 (4.3)	
Postoperative complications, n (%)						0.287*
	Non-postoperative complications		94 (88.7)	75 (90.4)	19 (82.6)	
	Having a postoperative complication		12 (11.3)	8 (9.6)	4 (17.4)	
		Surgical site infection	3 (2.8)	3 (3.6)	–	
		Pneumonia	1 (0.9)	–	1 (4.3)	
		Intraabdominal abscess	1 (0.9)	1 (1.2)	–	
		Thromboembolism	2 (1.9)	2 (2.4)	–	
		Urinary tract infection	1 (0.9)	1 (1.2)	–	
		Hematoma	1 (0.9)	–	1 (4.3)	
		Atelectasis	3 (2.8)	1 (1.2)	2 (8.7)	
Hospital stay (day), median (Q1–Q3)			2 (2–3.3)	2 (2–2)	4 (2–5)	**<0.001**

* Fisher's exact test was used.

SD: standard deviation; ASA: American Anesthesia Society. Bold values indicate statistically significant results where p<0.05.

In multivariate analysis, being operated on by a senior resident surgeon was not a risk factor for either postoperative complications or conversion to open surgery. Furthermore, in multivariate analysis, only male gender was found to be an independent risk factor for postoperative complications (OR 5.284; 95%CI 1.368–20.415; p=0.016) (Table 2).

**Table 2:** Univariate and multivariate logistic regression analysis of risk factors for postoperative complications and conversion to open surgery.

For complications	Univariate analyses	p-value	Multivariate analyses	p-value
	Odds ratio (95%CI)		Odds ratio (95%CI)	
Age	1.000 (0.951–1.051)	0.999	0.986 (0.928–1.049)	0.664
Male	**5.714 (1.611–20.268)**	**0.007**	**5.284 (1.368–20.415)**	**0.016**
Previous surgery	1.680 (0.321–8.780)	0.539	1.045 (0.165–6.606)	0.963
Resident surgery	1.974 (0.537–7.253)	0.306	1.236 (0.255–5.995)	0.793
Operation length	1.004 (0.985–1.023)	0.672	1.010 (0.987–1.035)	0.395
Mass diameter	0.985 (0.958–1.013)	0.284	0.986 (0.954–1.018)	0.378
Right side	0.431 (0.110–1.694)	0.228	0.558 (0.127–2.441)	0.438
Open surgery	0.764 (0.089–6.553)	0.806	0.551 (0.049–6.210)	0.629
**For open surgery**	**Univariate analyses**	**p-value**	**Multivariate analyses**	**p-value**
	**Odds ratio (95%CI)**		**Odds ratio (95%CI)**	
Age	0.987 (0.939–1.038)	0.612	0.962 (0.904–1.025)	0.229
Male	0.951 (0.189–4.783)	0.951	0.768 (0.133–4.441)	0.769
Previous surgery	1.889 (0.357–9.998)	0.454	2.108 (0.314–14.151)	0.443
Resident surgery	0.332 (0.040–2.737)	0.306	0.162 (0.015–1.694)	0.128
Operation length	1.008 (0.989–1.029)	0.407	1.019 (0.994–1.044)	0.135
Mass diameter	0.994 (0.968–1.021)	0.656	0.984 (0.955–1.013)	0.274
Right side	0.280 (0.057–1.368)	0.116	0.246 (0.047–1.293)	0.098

CI: confidence interval. Bold values indicate statistically significant results where p<0.05.

## DISCUSSION

Recent advancements in minimally invasive techniques and their growing replacement of open procedures have significantly increased the importance of advanced laparoscopic procedures in resident training. However, concerns have been raised about the safe and effective execution of complex laparoscopic techniques, such as LA, by residents. Despite its technical challenges and potential for serious morbidity, LA is generally considered a safe method^3,4^.

This study aimed to assess whether senior residents (4 years and above) can safely perform LA under the guidance of experienced surgeons. The results demonstrated that LA can indeed be safely performed by senior residents with appropriate supervision. Several other studies have reported similar findings. For instance, Dworak et al. compared outcomes in 54 patients operated on by residents and 246 patients operated on by senior surgeons. They found no statistically significant differences between the groups and concluded that LA could be safely performed by residents under supervision^5^. Similarly, Horesh et al. reached the same conclusion in a study conducted in 2016^6^.

Most experts suggest that performing 20–50 cases is required to master the learning curve for LA^3,7^. Achieving this volume necessitates training in high-volume centers^8^. Our department, a recognized reference center for endocrine and advanced laparoscopic surgery, offers this volume and provides comprehensive resident training in LA. While many other training hospitals in our country are reference centers for these procedures, they are often hesitant to include advanced laparoscopic techniques like LA in their resident training due to concerns about technical difficulty and potential morbidity.

Our goal is to encourage these training hospitals to integrate LA into their resident training programs by demonstrating that the procedure can be safely performed by senior residents under supervision. We believe hands-on training is essential for safe and effective learning. In our study, the length of hospital stay for patients operated on by residents was longer compared to those operated on by senior surgeons. This could be attributed to the increased caution exercised during postoperative care for patients treated by residents. Interestingly, Dworak et al. reported shorter hospital stays for patients operated on by residents, attributing this to the selection of simpler cases for trainees^5^.

When comparing mass diameters, Dworak et al. found no significant differences between the groups^5^. In our study, however, patients operated on by residents had statistically smaller masses, which aligns with the practice of selecting smaller, less complex cases in the early stages of the learning curve. Larger masses were reserved for senior surgeons.

In our study, male gender was identified as an independent risk factor for postoperative complications after laparoscopic adrenalectomy. While there are studies in the literature indicating a higher risk for the female gender, findings regarding gender are inconsistent. Brunaud et al. reported that female gender is not a risk factor for postoperative general morbidity, but it is a risk factor for cardiovascular morbidity^9^. Wang et al. added female gender as a risk factor in their nomogram to predict morbidity^10^. Phillips et al. also developed a nomogram for postoperative complications, but found that gender was not a risk factor in their calculations^11^. Parente et al. evaluated patients undergoing surgery for pheochromocytoma and, in their multivariate analysis, found that gender was not a risk factor for postoperative complications^12^. Although male gender was identified as a risk factor in our study, we found that male patients had higher ASA scores when we examined this factor. We believe this difference may have increased the risk of postoperative complications in male patients.

## CONCLUSION

In conclusion, LA can be safely performed by senior residents under the supervision of experienced surgeons. Selecting smaller masses for early-stage training is a prudent strategy to facilitate learning. LA training should be conducted in high-volume centers that serve as reference hubs for advanced laparoscopic and endocrine surgery.

## Data Availability

The datasets generated and/or analyzed during the current study are available from the corresponding author upon reasonable request.
